# Epidemiologic survey and genotyping of *Enterocytozoon bieneusi* in sika deer (*Cervus nippon*) in four provinces of China

**DOI:** 10.1051/parasite/2026049

**Published:** 2026-09-03

**Authors:** Hong-Di Zhuang, Wen-Tao Xiang, Ting Li, Bing-Bing Cui, Hai-Feng Feng, Jun-Fei Wang, Yi-Fan Zhang, Jia-Yu Yu, Qiu-Chi Jiang, Yu-Tao Chen, Jian-Ming Li, Nai-Chao Diao, Rui Du, Fei Liu

**Affiliations:** 1 College of Animal Science and Technology, Jilin Agricultural University Changchun 130118 PR China; 2 Shuangyang District Veterinary Drugs and Feed Supervision and Inspection Institute Changchun 130600 PR China; 3 Animal Health Supervision Institute of Jilin Province Changchun 130100 PR China; 4 Changchun Zoological and Botanical Garden Changchun 130022 PR China; 5 College of Chinese Medicinal Materials, Jilin Agricultural University Changchun 130118 PR China; 6 Department of Veterinary Medicine, College of Agriculture, Yanbian University Yanji 133002 PR China

**Keywords:** Sika deer, *Enterocytozoon bieneusi*, Zoonotic, Epidemiology, China

## Abstract

Microsporidia are obligate intracellular parasites with a wide host range. To elucidate the relationship between *Enterocytozoon bieneusi* infection dynamics and evolution within China’s sika deer population, 466 fresh fecal samples were collected from May to October 2024 across four provinces, Jilin, Liaoning, Heilongjiang, and Shandong. The ITS region of ribosomal RNA (rRNA) was amplified using nested PCR, enabling the identification and genotyping of *E. bieneusi.* The overall prevalence of *E. bieneusi* in sika deer was 44.4% (207/466). Province-specific prevalence was highest in Shandong (76.0%, 38/50) and Heilongjiang (75.3%, 67/89), followed by Jilin (38.7%, 65/168), and lowest in Liaoning (23.3%, 37/159). The prevalence in autumn (44.9%) was slightly higher than in summer (41.7%), and the prevalence in adult deer (48.7%) was found to be significantly higher than in young deer (31.6%). In this study, we identified a total of 11 *E. bieneusi* genotypes, including 9 known genotypes (BEB6, HND-II, LND-I, JLD-XIX, CHN-F1, HLJD-I, HLJD-III, Type IV, and COS-I) and 2 novel genotypes (named JL-D-4 and JL-D-5). Among them, BEB6 was the dominant genotype (38.7%, 80/207). This study enriches the genotype data of *E. bieneusi*, reveals the prevalence of *E. bieneusi* in some sika deer populations, and provides a reference for subsequent epidemiologic research as well as prevention and control efforts.

## Abstract (fr)

Les microsporidies sont des parasites intracellulaires obligatoires, avec un large spectre d'hôtes. Afin d'élucider la dynamique de l'infection par *Enterocytozoon bieneusi* et son évolution au sein des populations de cerfs sika en Chine, 466 échantillons de fèces fraîches ont été collectés entre mai et octobre 2024 dans quatre provinces : le Jilin, le Liaoning, le Heilongjiang et le Shandong. La région ITS de l'ARNr a été amplifiée par PCR nichée, permettant l'identification et le génotypage d'*E. bieneusi*. La prévalence globale d'*E. bieneusi* chez le cerf sika était de 44,4 % (207/466). Par province, la prévalence la plus élevée a été observée au Shandong (76,0 %, 38/50) et au Heilongjiang (75,3 %, 67/89), suivis du Jilin (38,7 %, 65/168), tandis que la plus faible a été relevée au Liaoning (23,3 %, 37/159). La prévalence en automne (44,9 %) était légèrement supérieure à celle observée en été (41,7 %), et la prévalence chez les cerfs adultes (48,7 %) était significativement plus élevée que chez les jeunes (31,6 %). Cette étude a permis d'identifier un total de 11 génotypes d'*E. bieneusi*, dont 9 génotypes connus (BEB6, HND-II, LND-I, JLD-XIX, CHN-F1, HLJD-I, HLJD-III, Type IV et COS-I) et 2 nouveaux génotypes (nommés JL-D-4 et JL-D-5). Parmi eux, le génotype BEB6 était prédominant (38,7 %, 80/207). Cette étude enrichit les données génotypiques relatives à *E. bieneusi*, révèle la prévalence de ce parasite au sein de certaines populations de cerfs sika et constitue une référence pour les recherches épidémiologiques ultérieures ainsi que pour les efforts de prévention et de lutte.

## Introduction

Microsporidia are a highly diverse group of obligate intracellular fungi, and some species are opportunistic pathogens with zoonotic potential [17]. *Enterocytozoon bieneusi* infects humans and a wide range of animals worldwide. In humans, *E. bieneusi* is clinically important: infections are often asymptomatic or self-limiting in immunocompetent individuals, but can cause severe, persistent diarrhea in immunocompromised patients, such as organ transplant recipients, people with advanced HIV infection, and those receiving immunomodulatory therapy [6, 21]. Transmission routes include ingestion of water or food, or contact with environmental surfaces contaminated with spores, or direct contact with an infected person [32]. Among the approximately 1,700 recognized microsporidian species spanning 220 genera [6], *E. bieneusi* has a wide host range and can infect humans, mammals, canines, rodents, and deer species (sika deer, water deer, and red deer), the latter of which has been identified as a major reservoir host [1, 10, 11, 20, 34].

The genetic diversity of *E. bieneusi* is complex and diverse. Analyses of the ITS region of ribosomal RNA (rRNA) have identified 820 distinct genotypes in at least 210 species of vertebrates (mammals, birds, reptiles, and amphibians) and invertebrates (insects and mussels) across more than 50 countries [5]. Based on host specificity and zoonotic potential, *E. bieneusi* genotypes are classified into phylogenetic groups (Group 1–15) [32]. Group 1 includes several zoonotic genotypes, such as D, Ebpc, and Peru 8 [16]. Group 2 genotypes primarily infect ruminants, but also include human-infecting genotypes BEB4, BEB6, I, and J [5]. Groups 3–15 are predominantly host-specific, but some genotypes (e.g., KIN-3 in Group 5, and Nig 3 and May 1 in Group 6) retain the ability to infect humans [9, 25].

China maintains the world’s largest population of farmed sika deer (*Cervus nippon*) and is the global leader in both the production and quality of deer antlers. Deer farming constitutes a significant economic activity in China. However, infection with *E. bieneusi* poses a threat to deer health and may adversely affect their growth and productivity [28]. In recent years, the prevalence and geographic range of *E. bieneusi* infection appear to be increasing, imposing a substantial economic burden on the deer farming industry. In view of this, we conducted this study as an epidemiologic investigation of *E. bieneusi* infections in the primary sika deer breeding regions of East China (Shandong Province) and Northeast China (Liaoning, Jilin, and Heilongjiang provinces). The findings aim to provide reference data for future prevention and control strategies against *E. bieneusi* infection in sika deer.

## Materials and methods

### Ethics statement

Sampling was carried out after obtaining written informed consent from the farm owners to collect samples. Fecal samples were collected non-invasively from the ground after natural defecation at deer farms. This study did not involve capturing, handling, sedating, hunting, euthanizing, or any experimental manipulation of live vertebrates. Therefore, this work did not constitute animal experimentation and did not require institutional animal ethics approval or a permit number. All procedures were conducted in accordance with relevant local regulations and institutional requirements.

### Sample collection

From May to October 2024, 466 fresh fecal samples were collected from sika deer farms in Shandong (*n* = 50), Liaoning (*n* = 159), Jilin (*n* = 168), and Heilongjiang (*n* = 89), China. During sampling, farm-management information was recorded through on-site observation and interviews with farm staff. The recorded variables included the number of farms sampled in each province, herd size, management/housing system, flooring type, enclosure area, group size per enclosure, stocking density, calculated as the group size of each enclosure divided by the corresponding enclosure area and expressed as deer/m^2^, manure removal frequency, and disinfection frequency. These farm characteristics are summarized in Supplementary Table S1. On-site observations and information from farm staff indicated that no other domestic animals were co-housed with the deer or kept in the immediate vicinity. Most sampled deer showed no apparent clinical signs; 13 had mild diarrhea, and a few of these also appeared emaciated. Samples were collected during morning inspections from freshly voided fecal piles at different time points. Only material from the center of each pile without visible soil contamination was collected. No more than three samples were collected per enclosure to reduce the likelihood of repeated sampling from the same deer. Sample information was recorded, and the samples were placed in bags, transported to the laboratory on dry ice, and stored at −80 °C.

### DNA extraction

Approximately 200 mg of each fecal sample was transferred into a 2 mL centrifuge tube containing 200 mg of glass beads. Genomic DNA was extracted using an E.Z.N.A.^®^ Stool DNA Kit (Shanghai Biotechnology Co., Ltd., Shanghai, China), according to the manufacturer’s instructions. DNA was eluted in 200 μL of elution buffer and stored at −20 °C until PCR amplification.

### Nested PCR amplification

Nested PCR was used to amplify an approximately 390 bp fragment of the rRNA internal transcribed spacer (ITS) region for the detection of *E. bieneusi* [12]. The primers ITS-F1 (5′–GGTCATAGGGATGAAGAG–3′) and ITS-R1 (5′–TTCGAGTTCTTTCGCGCTC–3′) were used for the primary PCR. ITS-F2 (5′–GCTCTGAATATCTATGGCT–3′) and ITS-R2 (5′–ATCGCCGACGGATCCAAGTG–3′) were used for the secondary PCR. Each PCR was performed in a total volume of 25 μL. The reaction mixture contained 12.5 μL of 2× PCR Mix (TaKaRa, Kusatsu, Japan), 8.5 μL of ddH_2_O, and 2 μL of template. It also contained 1 μL each of the forward and reverse primers (10 μM; final concentration, 0.4 μM each). Extracted DNA was used as the template for the primary PCR. For the secondary PCR, 2 μL of the undiluted primary PCR product was added directly as the template. Both PCR rounds were performed under the following conditions: initial denaturation at 94 °C for 5 min, followed by 35 amplification cycles. Each cycle consisted of denaturation at 94 °C for 45 s, annealing at 55 °C for 45 s, and extension at 72 °C for 1 min. A final extension was performed at 72 °C for 10 min. The secondary PCR products were separated by electrophoresis on 1.5% agarose gels and visualized using a QuickGel 6200 system (Monad Biotech Co., Ltd., Hubei, China).

### Sequence analysis

All *E. bieneusi-*positive PCR products were sent to Shanghai Biological Engineering Co., Ltd., for bidirectional sequencing. The ITS sequences were compared with reference sequences in the GenBank database using BLAST. CD-HIT (threshold = 0.99) was used to cluster sequences obtained in this study. PubMLST was used to identify *E. bieneusi* genotypes. All novel genotypes were named based on sequence features in the ITS region [22].

### Phylogenetic analysis and nucleotide sequence accession numbers

The ClustalW algorithm in MEGA11 was used to align 12 representative sequences from this study and 22 reference sequences from the GenBank. The phylogenetic analysis was conducted by neighbor-joining (NJ) method and the Kimura 2-parameter model. Additionally, 1,000 bootstrap replications were performed to assess the stability of the results.

The sequences generated in this study were deposited in GenBank under accession numbers PV298547–PV298558.

### Statistical analyses

The prevalence of *E. bieneusi* and the corresponding 95% confidence intervals (95% CIs) were calculated using the Wald method. Differences in prevalence among regions, seasons, and age groups were assessed using Pearson’s chi-square test in SAS, version 9.4. Univariable binary logistic regression was performed in SPSS Statistics, version 26.0 to estimate odds ratios (ORs) and their 95% CIs. Jilin, summer, and young deer were used as the reference categories. All tests were two-sided, and *p* < 0.05 was considered statistically significant.

## Results

### Infection status of *Enterocytozoon bieneusi* in sika deer

The overall prevalence of *E. bieneusi* in sika deer was 44.4% (207/466; 95% CI: 39.91–48.93). Five of the 13 diarrheic samples were positive (38.5%). Prevalence differed significantly among regions (χ^2^ = 85.571, df = 3, *p* < 0.0001), ranging from 23.3% in Liaoning Province to 76.0% in Shandong Province. No significant seasonal difference was observed (*p* = 0.609), although prevalence was slightly higher in autumn than in summer. Age was significantly associated with infection (χ^2^ = 10.362, df = 1, *p* = 0.0013), with adult deer showing a higher prevalence than young deer (48.7% vs. 31.6%) and approximately twice the odds of infection (OR = 2.05; 95% CI: 1.32–3.20) (Table 1).

**Table 1:** Prevalence of *Enterocytozoon bieneusi* infection in sika deer by region, season, and age.

Factor	Category	No. tested	No. positive	% (95% CI)	χ^2^/*df*/*P*	OR (95% CI)
Regions	Jilin	168	65	38.7 (31.19–46.19)	85.571/3/<0.0001	Reference
	Heilongjiang	89	67	75.3 (66.46–84.34)		4.83 (2.72–8.56)
	Liaoning	159	37	23.3 (16.49–30.05)		0.48 (0.30–0.78)
	Shandong	50	38	76.0 (64.12–87.88)		5.02 (2.44–10.30)
Season	Summer	72	30	41.7 (30.28–53.05)	0.262/1/0.609	Reference
	Autumn	394	177	44.9 (40.00–49.70)		1.14 (0.69–1.90)
Age	Young	117	37	31.6 (23.20–40.05)	10.362/1/0.0013	Reference
	Adult	349	170	48.7 (43.47–53.93)		2.05 (1.32–3.20)
Total	–	466	207	44.4 (39.91–48.93)	–	–

Province-stratified analyses are presented in Supplementary Table S2. At the overall level, no significant seasonal difference was detected, although prevalence was slightly higher in autumn than in summer. However, seasonal patterns varied among provinces: prevalence was higher in autumn than in summer in Jilin, Heilongjiang, and Shandong, but lower in autumn than in summer in Liaoning. The province with the highest prevalence differed between seasons, with Liaoning showing the highest prevalence in summer and Shandong in autumn; similarly, the lowest prevalence was observed in Jilin in summer and Liaoning in autumn. Age-related patterns were more consistent, with adult deer showing numerically higher prevalence than young deer in all four provinces, although the magnitude and statistical significance of this difference varied by province; this difference was most pronounced in Heilongjiang (92.1% vs. 34.6%) and minimal in Liaoning (23.4% vs. 22.9%).

### Genotype distribution

A total of 11 *E. bieneusi* genotypes were identified among the 207 positive samples, including nine known genotypes (BEB6, JLD-XIX, HND-II, CHN-F1, LND-I, HLJD-I, HLJD-III, Type IV, and COS-I) and two novel genotypes (JL-D-4 and JL-D-5). BEB6 was the dominant genotype, accounting for 38.7% (80/207) of all positive samples, followed by JLD-XIX (16.4%, 34/207) and HLJD-III (11.1%, 23/207). BEB6 was detected in all four provinces, whereas several genotypes showed province-specific distributions: COS-I, JL-D-4, and JL-D-5 were detected only in Jilin; HLJD-I and HLJD-III only in Heilongjiang; LND-I and CHN-F1 only in Liaoning; and HND-II and Type IV only in Shandong. The known zoonotic genotypes HND-II, Type IV, and CHN-F1 accounted for 4.8%, 3.9%, and 2.9% of positive samples, respectively (Table 2).

**Table 2:** Genotype distribution of *Enterocytozoon bieneusi* in sika deer in four provinces.

Factor	Category	Genotype
Regions	Jilin	BEB6 (*n* = 31), JLD-XIX (*n* = 20), COS-I (*n* = 12), JL-D-5 (*n* = 1), JL-D-4 (*n* = 1)
	Heilongjiang	BEB6 (*n* = 20), JLD-XIX (*n* = 6), HLJD-I (*n* = 18), HLJD-III (*n* = 23)
	Liaoning	BEB6 (*n* = 9), JLD-XIX (*n* = 8), LND-I (*n* = 14), CHN-F1 (*n* = 6)
	Shandong	BEB6 (*n* = 20), Type IV (*n* = 8), HND-II (*n* = 10)
Season	Summer	BEB6 (*n* = 10), JLD-XIX (*n* = 7), HND-II (*n* = 3), LND-I (*n* = 6), HLJD-I (*n* = 2), COS-I (*n* = 2)
	Autumn	BEB6 (*n* = 70), JLD-XIX (*n* = 27), HND-II (*n* = 7), LND-I (*n* = 8), HLJD-I (*n* = 16), HLJD-III (*n* = 23), CHN-F1 (*n* = 6), Type IV (*n* = 8), COS-I (*n* = 10), JL-D-4 (*n* = 1), JL-D-5 (*n* = 1)
Age	Adult	BEB6 (*n* = 70), JLD-XIX (*n* = 15), HND-II (*n* = 7), LND-I (*n* = 14), HLJD-I (*n* = 18), HLJD-III (*n* = 23), CHN-F1 (*n* = 4), Type IV (*n* = 6), COS-I (*n* = 12), JL-D-4 (*n* = 1)
	Young	BEB6 (*n* = 10), JLD-XIX (*n* = 19), HND-II (*n* = 3), CHN-F1 (*n* = 2), Type IV (*n* = 2), JL-D-5 (*n* = 1)
Total	–	BEB6 (*n* = 80), JLD-XIX (*n* = 34), HND-II (*n* = 10), CHN-F1 (*n* = 6), LND-I (*n* = 14), HLJD-I (*n* = 18), HLJD-III (*n* = 23), Type IV (*n* = 8), COS-I (*n* = 12), JL-D-4 (*n* = 1), JL-D-5 (*n* = 1)

### Genotyping and phylogenetic analyses

**Figure 1:**
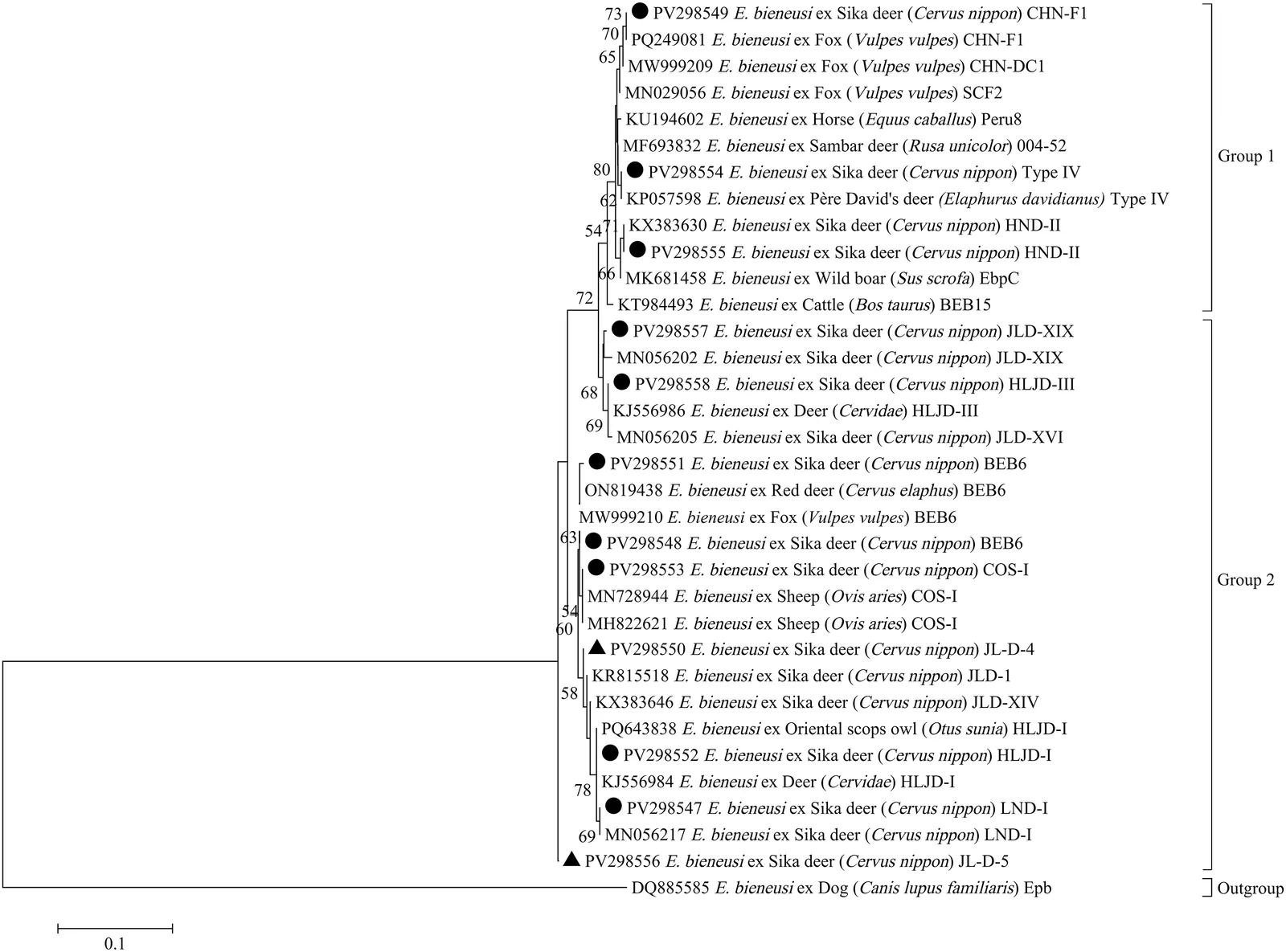
Neighbor-joining phylogenetic tree of *Enterocytozoon bieneusi* based on ITS sequences and constructed using the Kimura 2-parameter model. Bootstrap values (>50%) from 1,000 replicates are shown at the nodes. Circles indicate known genotypes, and triangles indicate novel genotypes identified in this study.

Phylogenetic analysis of the ITS sequences assigned the 11 *E. bieneusi* genotypes to Groups 1 and 2. The three known zoonotic genotypes, CHN-F1, Type IV, and HND-II, clustered within Group 1. The CHN-F1 sequence from sika deer (PV298549) grouped with fox-derived CHN-F1 (PQ249081) and CHN-DC1 (MW999209), whereas the Type IV sequence (PV298554) clustered with a Type IV sequence from Père David’s deer (KP057598). The remaining genotypes were placed in Group 2. Representative BEB6 sequences from this study clustered with BEB6 sequences from red deer and fox, and the COS-I sequence grouped with COS-I sequences from sheep. JLD-XIX, HLJD-I, HLJD-III, LND-I, and HND-II clustered with their corresponding reference genotypes. The two novel genotypes were also placed in Group 2: JL-D-4 (PV298550) was closely related to the sika deer-derived JLD-I and JLD-XIV genotypes, whereas JL-D-5 (PV298556) formed a distinct branch within Group 2 (Fig. 1).

## Discussion

In this study, *Enterocytozoon bieneusi* was detected in farmed sika deer from four provinces, with an overall prevalence of 44.4%. This prevalence was higher than most values previously reported in cervids from China. For example, previous studies reported a prevalence of 7.06% in sika deer from Jilin Province, as well as prevalences of 35.9% and 37.5% in sika deer and red deer, respectively, sampled from Henan and Jilin Provinces [7, 30]. Lower prevalences were also reported in red deer from Heilongjiang Province (6.8%) and Liaoning Province (8.3%) [33]. The prevalence observed in the present study was also higher than the 24.5% reported in Père David’s deer from Beijing [28]. In another study conducted in Beijing, prevalences of 12.5%, 30.0%, and 27.3% were reported in sika deer, Père David’s deer, and fallow deer, respectively [29].

Among cervids outside China, the prevalence observed in the present study also exceeded values reported in white-tailed deer from the United States (32.5%) and red deer from Spain (10.4%) [2, 24]. Compared with other animal hosts, the prevalence in sika deer in the present study was generally higher than that reported in horses from Korea (1.3%) [14], cattle from Austria (4.6%) [19], pet dogs from Yunnan Province, China (4.9%) [8], cats from Shanghai, China (5.6%) [27], farmed foxes from northern China (12.25%) [31], and cattle from Heilongjiang Province, China (30.1%) [35]. However, it was lower than the prevalence reported in pigs from Jilin Province (60.3%) [18]. These comparisons indicate that farmed sika deer may represent a potentially important host of *E. bieneusi*. Nevertheless, differences in host species, geographic location, husbandry practices, sampling period, sample size, and detection methods may have contributed to the variation in prevalence among studies.

Prevalence varied markedly among provinces (23.3%–76.0%), with higher values in Shandong and Heilongjiang than in Liaoning. This variation may be partly associated with differences in farm environment and management. All sampled farms used captive enclosed-pen housing systems and daily manure removal; however, earthen flooring was recorded in some farms in Shandong and Heilongjiang, the two provinces with the highest prevalence. Earthen flooring may more readily retain fecal contamination and moisture and may reduce the effectiveness of disinfection, thereby favoring the environmental persistence of *E. bieneusi* spores. Differences in enclosure area and stocking density may also influence contact opportunities among deer and the accumulation of fecal contamination in the farm environment. The lower disinfection frequency in Heilongjiang may have further contributed to its higher prevalence. However, Jilin and Liaoning showed different prevalences despite having similar recorded farm conditions, indicating that the recorded variables alone cannot fully explain the regional variation. Other unmeasured factors, including local climate, microenvironmental humidity, and broader environmental sanitation, warrant further investigation.

The overall prevalence of *E. bieneusi* was significantly higher in adult than in young sika deer (48.7% vs. 31.6%), consistent with previous findings from Heilongjiang, Jilin, Liaoning, and Inner Mongolia [26]. Province-stratified analysis also showed numerically higher prevalence in adults across all four provinces, although the magnitude of this age-related difference varied. This pattern may reflect cumulative exposure to *E. bieneusi* spores over time, while province-specific husbandry and environmental conditions may further modify age-related risk.

At the overall level, no significant seasonal difference was detected, although prevalence was slightly higher in autumn than in summer. However, province-stratified analysis suggested that seasonal patterns were not uniform across provinces, indicating that seasonal effects may be modified by local husbandry and environmental conditions.

In the present study, 11 ITS genotypes of *E. bieneusi* were identified in sika deer, with BEB6 being the dominant genotype, accounting for 38.7% (80/207) of the positive samples. Although BEB6 is classified in Group 2, previous studies have shown that this genotype has broad host adaptability and has been detected in humans, various livestock species, wild animals, and aquatic environments [3, 4, 12, 13, 15, 23]. These findings are partly consistent with previous studies from Jilin Province and other regions of northern China. An earlier study in sika deer in Jilin Province reported eight genotypes, among which genotype J was predominant, whereas BEB6 was detected at a lower frequency [30]. In contrast, a subsequent study of sika deer and red deer in Jilin and Henan provinces showed that BEB6 was the predominant genotype and identified several JLD- and HND-related genotypes [7]. A later study of dairy calves and sika deer in four provinces of northern China also reported a high frequency of BEB6 and detected several deer-associated genotypes, including HLJD-I, HLJD-III, and COS-I [26]. These differences suggest that the dominant genotypes and genotype composition of deer-derived *E. bieneusi* may be influenced by region, farm, or management conditions.

In addition to BEB6, relatively high numbers of JLD-XIX, HLJD-I, HLJD-III, and LND-I were detected in the present study. Among these genotypes, LND-I and JLD-XIX were first detected in sika deer from Liaoning and Jilin [26], whereas HLJD-I and HLJD-III were first reported in sika deer from Heilongjiang [34]. This further indicates that these deer-associated genotypes may continue to circulate in northeastern China. Notably, Type IV and HND-II were detected in samples from Shandong Province. Because Type IV has been reported in humans and various animals [16], its occurrence in farmed sika deer may have public health significance. In addition, the two novel genotypes, JL-D-4 and JL-D-5, clustered in Group 2, suggesting that they may have distinct host-adaptation characteristics. Overall, farmed sika deer may serve as important reservoir hosts for diverse *E. bieneusi* genotypes.

Although no other domestic animals were co-housed with the sampled deer or kept in the immediate vicinity, potential animal reservoirs, such as small carnivores, rodents, and wildlife, as well as potential environmental sources, including shared water sources, were not systematically investigated. Given the high prevalence of *E. bieneusi* in the sampled deer, further investigation of these potential reservoirs and environmental sources is warranted to clarify possible transmission routes, and to better assess the associated public health implications.

This study has three main limitations. First, the general health status of sampled animals was recorded mainly based on field observations and information provided by farm staff. However, systematic clinical examinations and standardized clinical scoring were not performed. Consequently, clinical signs such as diarrhea were not included in the risk-factor analysis. Second, sampling was conducted only during summer and autumn, which may not fully capture seasonal variation in *E. bieneusi* prevalence. Third, although basic farm-management variables, including stocking density, were recorded descriptively, these data were not incorporated into statistical risk-factor analyses because of the limited number of farms and the observational nature of the farm-level information. Future studies should include larger sample sizes, year-round surveillance, standardized clinical assessment, and systematically collected farm-management data to enable a more comprehensive epidemiologic assessment.

## Conclusion

In summary, the results of this study showed an overall *Enterocytozoon bieneusi* prevalence of 44.4% in farmed sika deer from four provinces in China. Eleven genotypes were identified among 207 positive samples. Notably, two novel genotypes, JL-D-4 and JL-D-5, were identified in sika deer, further expanding the known genetic diversity of *E. bieneusi* in this host. The detection of zoonotic genotypes belonging to Group 1 suggests that farmed sika deer may serve as potential reservoir hosts for zoonotic transmission. Therefore, routine screening for *E. bieneusi* and strengthened monitoring of farm environments are recommended to better assess and reduce potential transmission risks.

## Data Availability

The datasets presented in this study can be found in online repositories. The names of the repository/repositories and accession number(s) can be found at: https://www.ncbi.nlm.nih.gov/genbank/, PV298547–PV298558.

## Supplementary material

*Supplementary Table S1*: Farm characteristics recorded during sampling. *Supplementary Table S2*: Province-stratified prevalence of E*nterocytozoon bieneusi* in sika deer according to season and age. The supplementary material of this article is available at https://www.parasite-journal.org/10.1051/parasite/2026049/olm.
