# Supplementary material for: Epidemiologic survey and genotyping of Enterocytozoon bieneusi in sika deer (Cervus nippon) in four provinces of China
Source: Parasite. 2026 Sep 3;33:48. doi: 10.1051/parasite/2026049 (PMC13544796; doi:10.1051/parasite/2026049)
Supplement: Supplementary file 1 [file parasite-33-48-s1.pdf]

Supplementary Table S1. Farm characteristics recorded during sampling.

Province	No. of farms	Herd size (deer/farm)	Management and housing system	Flooring type (no. of farms)	Group size (deer/enclosure)	Enclosure area (m²/enclosure)	Stocking density (deer/m²)	Manure removal frequency	Disinfectio n frequency
Jilin	7	240–483	Captive enclosed pens	Concrete floor (7)	20–30	200–300	0.100	Once daily	2–3 times/week
Liaoning	7	120–512	Captive enclosed pens	Concrete floor (7)	20–38	200–380	0.100	Once daily	2–3 times/week
Heilongjiang	5	91–134	Captive enclosed pens	Concrete floor (2); earthen floor (3)	15–35	225–525	0.067	Once daily	1–2 times/week
Shandong	4	76–120	Captive enclosed pens	Concrete floor (2); earthen floor (2)	15–25	150–325	0.077–0.100	Once daily	2 times/week

Note. Stocking density was calculated as group size per enclosure divided by enclosure area and is expressed as deer/m². Farm management information was obtained through on-site observation and interviews with farm staff.
