# Supplementary material for: Epidemiologic survey and genotyping of Enterocytozoon bieneusi in sika deer (Cervus nippon) in four provinces of China
Source: Parasite. 2026 Sep 3;33:48. doi: 10.1051/parasite/2026049 (PMC13544796; doi:10.1051/parasite/2026049)
Supplement: Supplementary file 2 [file parasite-33-48-s2.pdf]

Supplementary Table S2. Province-stratified prevalence of *Enterocytozoon bieneusi* in sika deer according to season and age.

Factors	Category	No.tested	No.positive	% (95%CI)	χ^2/*df*/ /*P*	OR (95%CI)
Season	Jilin-Summer	25	9	36.0(17.18-54.82)	0.090/1/0.765	Reference
	Jilin-Autumn	143	56	39.2(31.16-47.16)		1.14(0.47-2.77)
	Heilongjiang-Summer	12	5	41.7(13.77-69.56)	8.422/1/0.0037	Reference
	Heilongjiang-Autumn	77	62	80.5(71.67-89.37)		5.79(1.61-20.79)
	Liaoning-Summer	25	12	48.0(28.42-67.58)	10.160/1/0.0014	Reference
	Liaoning-Autumn	134	25	18.7(12.06-25.25)		0.25(0.10-0.61)
	Shandong-Summer	10	4	40.0(9.64-70.36)	8.882/1/0.0029	Reference
	Shandong-Autumn	40	34	85.0(73.93-96.07)		8.50(1.83-39.42)
Age	Jilin-Young<18 months	40	11	27.5(13.66-41.34)	2.772/1/0.096	Reference
	Jilin- Adult ≥18 months	128	54	42.2(33.63-50.74)		1.92(0.88-4.19)
	Heilongjiang-Young<18 months	26	9	34.6(16.33-52.90)	32.641/1/<0.0001	Reference
	Heilongjiang- Adult ≥18 months	63	58	92.1(85.39-98.74)		21.91(6.47-74.19)
	Liaoning-Young<18 months	35	8	22.9(8.95-36.77)	0.004/1/0.948	Reference
	Liaoning- Adult ≥18 months	124	29	23.4(15.94-30.84)		1.03(0.42-2.51)
	Shandong-Young<18 months	16	9	56.3(31.94-80.56)	5.032/1/0.025	Reference
	Shandong-Adult ≥18 months	34	29	85.3(73.39-97.20)		4.51(1.15-17.75)
Total	-	466	207	44.4(39.91-48.93)	-	-

Note: Young deer were defined as <18 months and adults as ≥18 months. Summer and young deer were used as reference categories for stratified comparisons. No winter samples were collected.
